# Working intentions of medical students in response to healthcare workplace violence and descending resources reform in China

**DOI:** 10.1186/s12909-022-03428-1

**Published:** 2022-05-09

**Authors:** Shuhong Wang, Hongjun Zhao, Zesheng Sun

**Affiliations:** 1 Department of Stomatology, Songjiang Hospital Affiliated to Shanghai Jiaotong University School of Medicine (Preparatory Stage), Shanghai, China; 2 School of Finance and Business, Shanghai Normal University, No. 100, Guilin Road, Xuhui District, Shanghai, 200234 China

**Keywords:** Career choice, Health care reform, Workplace violence, Medical students, Cross-sectional study, Ordered logit model

## Abstract

**Background:**

In order to curb healthcare workplace violence (WPV) and better allocate healthcare resources, China launched the descending resources reform in 2013 and tightened the anti-violence legal environment simultaneously. Medical students are expected to reconsider their working intentions of entering the medical market (inter-market effect) and choosing high- or low-level hospitals (intra-market effect) in response to the evolving WPV. The goal of this study was to explore the link between the perceived WPV incidence and medical students’ willingness to practice medicine in the context of China’s descending resources reform.

**Method:**

Medical students were selected with cluster sampling from 8 medical colleges in Zhejiang Province, China, and 1497 valid questionnaires were collected by using a five-point unbalanced scale, to perform cross-sectional empirical research using the ordered logit model (OLM).

**Results:**

The perceived WPV incidence negatively correlate with the willingness of medical students to practice medicine but positively correlate with their willingness to practice in low-level hospitals, indicating the existence of inter- and intra-market effects. The anti-violence legal environment has no direct link with working intention but contributes to the perceived decline in the incidence of violence. Descending resources reform has simultaneous opposite effects on medical students, with the coexistence of prudent motives driven by reform costs and optimistic expectations of sharing external benefits.

**Conclusions:**

Safety needs and risk aversion motive play an important role in medical students’ career choice when facing severe WPV. Tightening of the anti-violence legal environment and the descending resources reform could drive medical students to low-level hospitals.

**Supplementary Information:**

The online version contains supplementary material available at 10.1186/s12909-022-03428-1.

## Introduction

Over the past two decades, the uneven allocation of resources in China’s medical market and resulting healthcare workplace violence (WPV) have received widespread attention in academia and politics [1–3]. Since 2013, China has promoted descending resources reform to reallocate health resources from high-level (tertiary) to low-level (primary) hospitals, i.e., the government forces and/or subsidizes doctors from high-level hospitals to move down to low-level hospitals, to improve the latter’s diagnostic and treatment capabilities through human capital spillover, thus attracting more patients [4]. In addition, since 2014, the high incidence of WPV has impelled the Chinese government to enact legislation and enforcement efforts to curb such behavior [5]. WPV would drive healthcare workers and medical students to leave health labor market, or seek jobs with lower risk and/or higher income among different level hospitals. Meanwhile, descending resources reform is designed to rebalance the healthcare market [6], and improvement in the legal environment is expected to curb WPV, thus influencing the willingness of medical students to practice medicine. This paper is the first to explore the link between WPV and medical students’ willingness to practice medicine in the context of China’s descending resources reform.

Recruiting and retaining medical students in areas of workforce need is of international concern. One branch of research tends to focus on the perspectives of employers [7, 8], i.e., approaches for selecting and recruiting medical students, while another branch tends to concentrate on factors influencing career choice from the perspective of medical students. In addition to factors such as parental influence, personality, medical education, and career advice and guidance [9–11], WPV and working intention responses of medical students have received increasing attention [12–14]. WPV is a widespread phenomenon that affects medical students and impedes future retention rates [12], resulting in negative career choice satisfaction and exit from the healthcare profession [13, 15]. Research suggests that there may be a direct link between episodes of violence and poor recruitment and retention rates [10], and that intense doctor-patient relationships can impact career choice among medical students, leading to an increase in students switching to other majors with lower violence risk, e.g., auxiliary medical departments [16]. However, more empirical evidence is needed to better understand the responses of medical students to WPV.

In a two-stage career selection process, i.e., high-school student--medical student--healthcare worker, practicing medicine results in higher factor specificity compared with other occupations [17]. In the context of deteriorating doctor-patient relationships and increasing WPV incidence in China, a decline in the willingness to study medicine has been reported in high-school students, leading to the lowering of admission scores and quality of students entering medical schools [18, 19]. Hu and Ye et al. separately reported that 94.61% of respondents and > 70% of doctors do not want their children to study medicine [20, 21]. WPV not only negatively impacts the intention of students to practice medicine, but also increases the intention of doctors to leave their jobs [22, 23]. If medical students decide to enter healthcare labor market, their choices are impacted by working conditions, salary, and other factors [24]. Medical students are more inclined to work in cities [25–28] and at higher-level hospitals that have better working conditions and higher salaries [29, 30]. Nevertheless, doctor-patient relationships and safety conditions are much better in low-level hospitals due to the lower incidence of violence [31, 32]. To date, however, very limited empirical studies have discussed medical students’ risk aversion effects of job-seeking.

The core research question is the working intention responses of medical students to WPV. Given other conditions, it could be assumed that, for the sake of personal safety, medical students may consider leaving the healthcare labor market or seeking employment in workplaces with lower safety risk. However, to identify the marginal effects of WPV on career choice, we must control for the effects of other policy variables that may influence medical student career choice. In this paper, we explored two main policy variables, i.e., changes in anti-violence laws and introduction of descending resources reform. Notably, changes in the anti-violence legal environment may affect medical student perception of the incidence of WPV and the degree to which their safety needs are fulfilled. Furthermore, the expected costs and benefits of descending resources reform in China, which asks young doctors from high-level hospitals to move to low-level or rural hospitals for several months or years, may also impact the career choices of medical students.

In this paper, we recognized that medical students will choose if they would enter healthcare labor market or not, and decide which level of hospitals they will choose to work as the response of WPV. Compared with previous research, this paper used questionnaire data to estimate the link between WPV and the working intentions of medical students in the context of the latest health resource reallocation policies. We also investigated WPV and the legal environment to estimate their impacts on the intention of medical students to practice medicine. This research provides evidence on medical students’ response to the descending resources reform, healthcare workplace violence, and the anti-violence legal environment of China in recent years.

## Background

### Descending resources reform

China’s public hospitals are divided into three grades. Grade one and two hospitals are usually owned by the government at or below the county level, and are defined as low-level hospitals; while tertiary grade hospitals are usually located in major cities, and are defined as high-level hospitals [4]. Prior to 2009, since the Chinese government reduced its investment and medical insurance coverage, the unbalanced allocation of health resources led to serious inequality, doctor-patient conflicts, and harmful social consequences. In 2009, new healthcare reform was established with a focus on abolishing medicine markups and adjusting medical service fees to correct distorted incentives for over-prescription and over-examination in response to public complaints about excessive medical costs [33].

However, the 2009 reform did not affect patient preference for high-level hospitals, and thus high-level hospital congestion and low-level hospital idleness remained problematic [34]. There are two main explanations: The first is that past investment focused on infrastructure rather than human capital; the treatment ability of low-level hospitals was not improved [3, 33], so low-level hospitals failed to address the discrepancy in human capital with high-level hospitals and win back patient trust. The second is that past reforms mainly impacted patient affordability from the perspective of demand, but not biased resource allocation or choice of care providers from the perspective of supply, thus medical students were reluctant to seek jobs at low-level hospitals. Therefore, the under-utilization and reduced capacity of low-level hospitals remain important challenges in China [34].

**Fig. 1:**
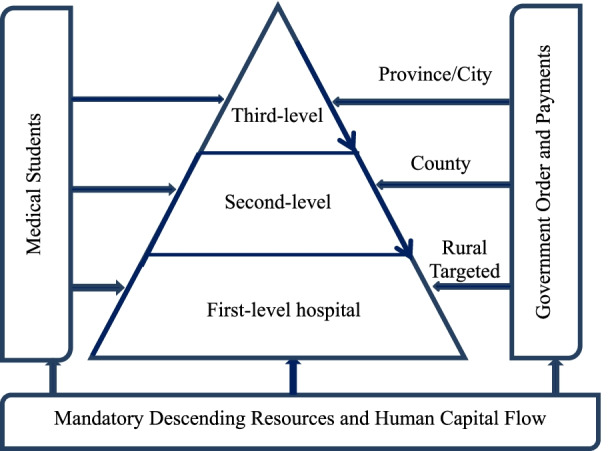
Descending resources reform and intended incentive structure

In 2013, the descending health resources reform was launched in Zhejiang, a pilot province in China undergoing comprehensive medical improvement. In 2014, the Chinese President, Xi Jinping, bolstered the measures implemented in this reform [35]. In subsequent years, this reform has gradually expanded to other pilot provinces and has become one of the core health policies of China for the reallocation of medical resources between rural (county and town) and urban (major city) areas. As provincial governments in China own both high-level and low-level hospitals, this reform mandatorily requests that doctors from high-level hospitals move to low-level hospitals for a certain period (usually a few months to 1 ~ 2 years), and asks for high-level hospitals to establish inter-hospital cooperative ties with low-level hospitals. At the same time, the government encourages medical students to work at low-level hospitals through targeted-area student policies and employment subsidies (Fig.1). The government also provides subsidies for all hospitals involved in the reform of medical resources reallocation.

According to the Zhejiang government [36], descending health resources reform can be summarized as follows. Firstly, for high-level hospitals, all province-level general hospitals/specialty hospitals should establish comprehensive trusteeships and ties with 2-4 county-level hospitals. No less than 5% of qualified doctors with intermediate or above professional titles should move to low-level hospitals. More than 80% of reallocated doctors should have intermediate or above professional titles. Secondly, for medical students, no less than 1000 targeted-area students should be trained to work in low-level hospitals each year. Low-level hospitals should recruit 10,000 medical students per year. Lastly, the government would provide financial subsidies for all level hospitals involved in the reform to (at least partially) compensate for their reform costs, and also provide tuition compensation for medical students who join low-level hospitals and facilitate income increase and job promotion.

### Congestion in China’s medical market and healthcare workplace violence

Sun et al. provided a detailed description of the congestion in the Chinese medical market [17], describing the coexistence of overcrowding in high-level city hospitals and underutilization of resources in low-level hospitals. This structural congestion has contributed to the long-term decline in treatment satisfaction and deterioration of the doctor-patient relationship, which have, in turn, evolved into an escalation in medical conflicts and frequent violence against healthcare workers. However, doctor-patient conflicts and healthcare workplace violence are unevenly distributed in the medical market: high-level hospitals with congestion suffer from higher dissatisfaction and incidences of disputes and violence [31], whereas low-level hospitals experience low resource utilization and limited violence because they can transfer high-risk patients to high-level hospitals.

Healthcare workplace violence is a global concern and affects healthcare workers and even medical students in nearly all health-care settings [37–39], however, the scale, frequency, and viciousness of attacks on healthcare workers in China are particularly severe [2]. One survey found that more than 67% of violent incidences reported by the media from 2000 to 2015 were related to tertiary (high-level) hospitals, with low-level hospitals experiencing lower incidences of violence [32]. Furthermore, more than 50% of violent events resulting in casualties in 2015-2016 occurred in tertiary hospitals and more than 40% occurred in emergency services, which were mainly caused by congestion rather than triggered by therapeutic effects [31]. In addition, previous research has stated that violent events in hospitals are often unreported, with tertiary hospitals having the highest tolerance rate for unreported violence [40]. It is also reported that 18.5% medical students have witnessed healthcare workplace violence [41]; most medical students receive violence-related information from hospital and news media and give negative responses [42, 43].

The serious healthcare workplace violence disclosed by the media impelled the Chinese government to issue the *Opinions on Punishing Medical-related Crimes and Maintaining Order in Hospitals* in 2014, which demanded the strengthening of violence prevention and control, as well as more severe punishment for perpetrators. In 2020, China’s top legislator approved comprehensive laws for protecting healthcare workers, which ban any organization or individual from threatening or harming healthcare workers. However, whether China has experienced an improvement in doctor-patient relationships and the incidence of healthcare workplace violence in the context of the current descending resources reform and anti-violence legal environment remains unclear. Thus, in the current study, we explored the link between healthcare workplace violence and medical students’ working intentions in the context of the latest reform and anti-violence legal environment.

## Methods

### Theory and hypotheses

We used Maslow’s hierarchy of needs theory as the core theory of this paper. According to this concept, humans have five basic needs in a hierarchical form from low to high, i.e., physiology, security, society, esteem, and self-actualization [44]. Medical students’ willingness to practice medicine is a labor market choice behavior associated with their expected cost-benefit analysis when making career choices. This analysis is determined by the expected satisfaction of different levels of needs brought by medical practitioners [45].

**Fig. 2:**
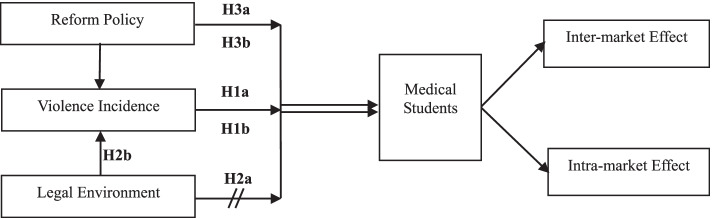
Conceptual model of medical students’ working intention

The conceptual models and hypotheses of this study are shown in Fig. 2. Based on China’s long-term policies in medical-price control and the government’s dominant role in hospital systems, certain degrees of satisfaction of Maslow’s hierarchy of needs (physiological, social, esteem, and self-actualization) can be treated as given. Since the incidence of violence regarding safety needs changes dynamically, medical students would reconsider their labor market choice by using all available violence-related information. As income cannot compensate for high violence risk, if the incidence of healthcare workplace violence is low or similar to the average experienced in different workplaces, it should not affect the willingness to practice medicine; however, if it is far beyond the average level, it should have a negative impact on the willingness to practice medicine (Hypothesis 1a, H1a; shown in Fig. 2). This negative impact can worsen due to the specificity of factors [4], which refers to the inflexibility to switch to other industries or occupations because of occupational lock-up after medical training. In this paper, we divided employers into two categories, i.e., high-level and low-level hospitals: if students realize that violence mainly occurs in high-level hospitals, their marginal willingness to work in low-level hospitals may increase compared to their willingness to work in high-level hospitals (Hypothesis 1b, H1b).

- **Hypothesis 1a**: Increased incidence of healthcare workplace violence reduces medical students’ willingness to practice medicine (inter-market effect).
- **Hypothesis 1b**: Increased incidence of healthcare workplace violence increases medical students’ willingness to work in low-level hospitals (intra-market effect).

The anti-violence legal environment involves *ex ante* and *ex post* levels and includes government, legislation, law enforcement, and hospital-related factors. The function of government in dealing with healthcare workplace violence involves two aspects, i.e., *ex-ante* prevention/protection and *ex-post* response. The role of hospitals is limited to *ex-ante* prevention. Regarding legislation and law enforcement, practitioners can evaluate whether current punishments for violent perpetrators are appropriate or not, whereas law enforcement is usually involved in *ex-post* responses after events. However, the above anti-violence legal environment is more likely to be an indirect indicator and intermediate variable affecting medical students’ willingness to practice medicine (Hypothesis 2a, H2a). The direct indicator is the effect of the existing legal environment, i.e., incidence of and trends in healthcare workplace violence, which directly affect medical student satisfaction with safety needs (Hypothesis 2b, H2b).

- **Hypothesis 2a**: Anti-violence legal environment does not affect medical students’ willingness to practice medicine.
- **Hypothesis 2b**: Anti-violence legal environment reduces incidence of healthcare workplace violence.

Healthcare workplace violence and the anti-violence legal environment interact with the exogenous constraints of existing health policies. Although concern may arise whether medical students are aware of the reform or not, we believe that students will use all available information to understand and respond to the reform. So, descending resources reform may involve two policy effects. The first is a negative effect on medical students who wish to practice medicine (Hypothesis 3a, H3a). The main reason is that doctors from high-level hospitals are required to move to low-level hospitals and to bear the reform costs. The usual practice is that they must leave in-service hospitals and workplaces for a few months to 1 year and work in low-level hospitals far from the city; young doctors are also often the ones that are reallocated, thus increasing their cost of living and causing inconvenience. Student awareness of this policy may weaken their willingness to practice medicine. As reform could help to alleviate congestion in high-level hospitals and idle resources in low-level hospitals, the external benefit of reform could be shared by students and trigger their willingness to practice medicine (Hypothesis 3b, H3b).

- **Hypothesis 3a**: Awareness of descending resources reform has a negative impact on medical students’ willingness to practice medicine.
- **Hypothesis 3b**: The realization of the goal of descending resources reform has a positive impact on medical students’ willingness to practice medicine.

### Questionnaire design and variables

Based on Maslow’s hierarchy of needs theory, a five-point unbalanced scale was used in this paper. In addition to demographic items, variables on working intention, reform cognition, working environment, and policy evaluation were included in the questionnaire (Table 1). In our research, working intention included the desire to practice medicine or to practice medicine in low-level hospitals after reform, which were used to measure inter- and intra-market effects, respectively.

**Table 1:** Questionnaire design, variables, and definitions

Category	Question item	Variable	Definition
Working intention	*Your desire to practice medicine after reform*	*Y*	1 decrease, 2 no change, 3–5 slight, significant, and very significant increase, respectively
	*Your desire to practice medicine in low-level hospitals after reform*	*Y1*	1–5 variables defined as *Y*
Demography	*Your education level*	*Education*	1 technical degree, 2–5 college, bachelor, Master, and PhD degree, respectively
	*Your grade*	*Grade*	1–5 variables for different grades
	*Are you a targeted-area student*	*Target*	1 for *Yes*, 0 for *No*
Working environment	*Your perceived level of respect for doctors*	*Respect*	1–5 very low, low, moderate, high, and very high, respectively
	*Your frequency of concern about violence*	*Concern*	1 never, 2 rarely, 3–5 occasional, frequent, and high, respectively
	*Your perceived incidence of violence*	*Violence*	1–5 very low, low, moderate, high, and very high, respectively
	*Your perceived improvement in incidence of violence after reform*	*Y2*	1–5 very low, low, moderate, high, and very high, respectively
Policy	*Your degree of recognition of reform*	*Recogn*	1–5 very low, low, moderate, high, and very high, respectively
	*Perceived policy effect*	*Policy*	1-5 negative, no effect, positive, good, and very good, respectively
	*health policy or anti-violence lesson*	*Lesson*	1 for *Yes*, 0 for *No*
Legal environment	*Government’s violence prevention*	*Legal*_*_A*_	1–5 very weak, weak, moderate, strong, and very strong, respectively
	*Government’s response to violence*	*Legal*_*_B*_	1–5 variables defined as *Legal*_*_A*_
	*Legislative punishment for violence*	*Legal*_*_C*_	1–5 variables defined as *Legal*_*_A*_
	*Anti-violence law enforcement*	*Legal*_*_D*_	1–5 variables defined as *Legal*_*_A*_
	*Hospitals’ protection for medical staff*	*Legal*_*_E*_	1–5 variables defined as *Legal*_*_A*_

The working environment was first measured based on the perceived level of respect for doctors and then violence-related items, which included frequency of concern about violence, perceived incidence of violence, and perceived improvement in incidence of violence after reform. For reform policy, four items were included: degree of recognition of reform, perceived policy effect, health policy, and anti-violence education. The anti-violence legal environment was measured based on government efforts, legislative punishment, law enforcement, and hospital protection.

Before the survey, the questionnaire used in this paper has experienced pre-release, expert review and revision. The survey was performed from August 2018 to September 2019. Medical students were selected with cluster sampling from 8 medical schools in different cities of Zhejiang Province, China. And all respondents provided informed consent to participate. We obtained 1641 questionnaires, of which 1497 were valid (effective rate of 97.33%).

Due to the differences among question items, we first set the different evaluation items from low to high and then converted them to 1–5 variables and converted questions with responses of only *Yes/No* to 1–0 variables. The correlation coefficients between measurement variables were all lower than 0.60. By using SPSS 23.0 and reliability tests, the Cronbach’s α coefficient was 0.804; and the Kaiser-Meyer-Olkin (KMO) value was 0.925, with Sig. = 0.000 for the Bartlett spheroid test, indicating that the scale and data were suitable for empirical analysis.

### Empirical model

Based on the above theory and hypotheses, we used *Violence* and *Legal* variables to depict impact on safety needs and corresponding legal environment changes, respectively. The variable *Respect* was used to measure the effect of esteem needs. The variable *Policy* was used to incorporate other needs in Maslow’s theory. As targeted-area students all come from rural (county and town) areas, resulting in a high correlation between the student origin (urban/rural) and *Target* variables; meanwhile, *grade* and age also has high correlation, we did not include student origin and age in the empirical model. The demographic variables (*Demographic*_*i*_) included *Target*, *Education*, and *Grade*. Health education, *Recog*, and *Concern* were also added to the model as control variables (*Control*_*k*_). The empirical model is as follows:

1 $$Y=C+{\sum}_i{Demopgraphic}_i+\beta Violence+\phi \operatorname{Re} spect+\phi Legal+\gamma Policy+{\sum}_k{\eta}_k{Control}_k$$

As the questionnaire data were discrete and most variables were ordered one, the ordered logit model (OLM) was utilized to estimate the model [46]. To test the robustness of the regression results, we used stepwise regression technology, and also chose different legal environment variables for empirical testing. All methods were carried out in accordance with relevant guidelines and regulations.

## Results

### Descriptive analysis

Table 2 shows the demographic distribution of samples. Undergraduate students and non-targeted students constituted the largest proportions, accounting for 79.09 and 94.99%, respectively. Students from urban areas and non-Zhejiang provinces accounted for 39.21 and 33.27%, respectively. Student participants ranged from 14.83% in the first year to 24.25% in the fourth year.

**Table 2:** Demographic distribution (sample size = 1497)

Student origin	Number (%)	Education level	Number (%)	Grade	Number (%)	Targeted-area student	Number (%)
Urban area	587 (39.21)	Technical	1 (0.07)	One	222 (14.83)	Yes	75 (5.01)
Rural area	910 (60.79)	College	18 (1.20)	Two	308 (20.57)	No	1422 (94.99)
		Bachelor	1184 (79.09)	Three	357 (23.85)		
Zhejiang Province	999 (66.73)	Master	251 (16.77)	Four	363 (24.25)		
Other provinces	498 (33.27)	Ph.D.	46 (3.07)	Five	247 (16.50)		

As shown in Table 3, the perceived incidence of violence was high, with scores of *very high* (5) and *high* (4) accounting for 64.67% of the total sample. In total, 41.82% of respondents chose *very high* as their perceived incidence of violence, whereas only 11.55% believed the incidence of violence to be low. However, respondent *Concern* was not high, with only 31.93% of participants paying *high* or *frequent* attention to violence and 50.43% of participants paying *occasional* attention to violence. Furthermore, 41.35% of respondents chose the *very low* (score of 1) recognition option for reform, with 37.74% of respondents believing that they knew something about this issue; 47.53% of respondents did not have any health policy course/knowledge and 60.39% of respondents received no anti-violence education.

**Table 3:** Evaluation and health education of violence and reform (%)

Evaluation	*Violence*	*Concern*	*Recogn*	Education	Policy	Anti-violence
5	41.82	11.56	6.28	Course	8.45	7.01
4	22.85	20.37	12.42	Teacher	19.02	13.05
3	24.11	50.43	19.04	Lecture	19.37	20.05
2	7.01	13.63	21.24	Self-learn	5.63	6.46
1	4.54	4.34	41.35	No	47.53	53.43

5 is highest score of evaluation and 1 is lowest

According to Table 4, we divided legal environment and policy evaluations into positive (score of 4-5), neutral (score of 3), and negative (1-2). The positive and negative evaluations of the legal environment were 18–20% and 46–54%, respectively. For reform, 56.24% of respondents gave a positive evaluation (3-5), with the remaining respondents giving a neutral (score of 2, 39.01%) or negative (1) evaluation.

**Table 4:** Evaluation of anti-violence legal environment and reform (%)

Evaluation	*Legal*_*_A*_	*Legal*_*_B*_	*Legal*_*_C*_	*Legal*_*_D*_	*Legal*_*_E*_	*Policy*
5	5.34	6.95	7.55	7.15	5.68	8.28
4	12.89	11.22	10.62	11.22	14.76	17.43
3	28.39	30.33	29.93	33.27	33.27	31.20
2	45.29	45.49	44.36	41.75	40.41	39.01
1	8.08	6.01	7.55	6.48	5.88	4.07

5 is highest score of evaluation and 1 is lowest

### Working intention response to health labor market

Test result of H1a. As seen in Table 5, *Violence* negatively affected willingness to practice medicine at the 1% significance level with robust results, thus confirming Hypothesis 1a. Results showed that healthcare workplace violence, as an exogenous variable that negatively influences safety needs, was not conducive to the choice to practice medicine. The variable *Respect*, which represents the esteem needs of medical students, did not significantly impact the willingness to practice medicine. This indicates that respect for healthcare workers is not essential for working intention.

**Table 5:** Estimation results of OLM regression of medical students’ working intention

Dependent variable: working intention Y (1 for decrease, 2 for no change, 3-5 for slight increase, significant increase, and great increase)					
Variable	Model 1: Legal__A_	Model 2: Legal__B_	Model 3: Legal__C_	Model 4: Legal__D_	Model 5: Legal__E_
Target	0.8102^***^ (0.2150)	0.7876^***^ (0.2154)	0.7709^***^ (0.2156)	0.7776^***^ (0.2149)	0.7932^***^ (0.2146)
Education	0.6571^***^ (0.1031)	0.6532^***^ (0.1031)	0.6572^***^ (0.1029)	0.6499^***^ (0.1032)	0.6327^***^ (0.1032)
Grade	−0.0934^**^ (0.0373)	− 0.0890^**^ (0.0373)	− 0.0864^**^ (0.0373)	− 0.0878^**^ (0.0372)	− 0.0870^**^ (0.0372)
Violence	− 0.4389^***^ (0.0456)	− 0.4364^***^ (0.0457)	− 0.4342^***^ (0.0457)	− 0.4355^***^ (0.0456)	− 0.4320^***^ (0.0457)
Concern	0.2329^***^ (0.0558)	0.2306^***^ (0.0559)	0.2291^***^ (0.0559)	0.2273^***^ (0.0560)	0.2160^***^ (0.0562)
Lesson	−0.1518 (0.1060)	− 0.1560 (0.1059)	− 0.1608 (0.1060)	−0.1683 (0.1064)	− 0.1733 (0.1062)
Respect	0.0186 (0.0622)	0.0071 (0.0613)	−0.0038 (0.0617)	0.0005 (0.0614)	−0.0213 (0.0621)
Legal	−0.0166 (0.0548)	0.0434 (0.0527)	0.0835 (0.0535)	0.0808 (0.0545)	0.1537^***^ (0.0575)
Recogn	−0.1322^***^ (0.0499)	−0.1345^***^ (0.0424)	− 0.1392^***^ (0.0425)	−0.1382^***^ (0.0425)	− 0.1453^***^ (0.0426)
Policy	0.2269^***^ (0.0545)	0.2202^***^ (0.0540)	0.2103^***^ (0.0545)	0.2090^**^ (0.0548)	0.2055^**^ (0.0542)
LR stat	320.6237^***^	321.2077^***^	322.9627^***^	322.7065^***^	327.6668^***^
Obs	1497	1497	1497	1497	1497

[1] ^***^,^**^ and^*^ denote significance level of 1, 5 and 10%, respectively; [2] Clustered standard error in parentheses

Test result of H2a. The variable *Legal* also had no significant impact on the willingness to practice medicine. These results provide evidence for Hypothesis 2a, i.e., that medical students treat the incidence of violence as the determinant factor affecting working intention, with the legal environment being less important. To test the robustness of the above results, we introduced five different legal environment variables to model 1-5, respectively. It is found that Hypothesis 2a could be supported consistently and robustly.

Test results of H3a and H3b. The variable *Policy* had a significantly positive effect on the intention to practice medicine; however, *Recogn* had a significantly negative impact, fully supporting Hypotheses 3a and 3b. These results demonstrate that the direct costs and external benefits of reform coexist and are recognized by medical students. Therefore, improvement in recognition failed to enhance the willingness to practice medicine.

For health policy education and demographic variables, our results implied that targeted-area medical students had significantly stronger willingness than that of non-targeted students. That is, cost-locking based on contracts had an incentive effect on the desire to practice medicine. We also found that the higher the level of education, the stronger was the willingness to practice medicine. This result shows that higher level education increases factor specificity and opportunity costs, and thus a higher intention to practice medicine would be rational. The variable *Grade* was significantly negative, suggesting that, under a certain education level, the higher the grade, the higher the risk of the choice behavior.

We reported two different regression results to test the robustness. Five different legal environment variables are introduced into the empirical models in Table 5, and then stepwise estimation results were reported in Table A1. All results verified the robustness of the above test results. Besides, the demographic variables *Target*, *Education*, and *Grade* all had the same significant impact. The results of health policy education were non-significant, indicating that health policy education does not link to the working intention directly.

### Working intention response to low-level hospitals

Test result of H1b. As shown in Table 6, the perceived incidence of violence had a positive impact on the desire to work in low-level hospitals, fully supporting the existence of intra-market effect shown in Hypothesis 1b. That is, considering the constraints of factor specificity and differences in the incidence of violence among different level hospitals, it is rational to choose to work in low-level hospitals to better satisfy safety needs.

**Table 6:** OLM estimation results of medical students’ intention to work in low-level hospitals

Dependent variable: working intention for low-level hospital Y1 (1 for decrease, 2 for no change, 3-5 for slight increase, significant increase, and great increase)					
Variable	Model 1: Legal__A_	Model 2: Legal__B_	Model 3: Legal__C_	Model 4: Legal__D_	Model 5: Legal__E_
Target	0.7949^***^ (0.2242)	0.7826^***^ (0.2235)	0.7884^***^ (0.2244)	0.8199^***^ (0.2247)	0.8796^***^ (0.2242)
Education	0.4278^***^ (0.1062)	0.4082^***^ (0.1062)	0.4178^***^ (0.1066)	0.4109^***^ (0.1061)	0.3736^***^ (0.1071)
Grade	0.0165 (0.0384)	0.0084 (0.0383)	0.0038 (0.0383)	0.0031 (0.0382)	−0.0127 (0.0380)
Violence	0.1285^***^ (0.0449)	0.1230^***^ (0.0448)	0.1223^***^(0.0448)	0.1195^***^ (0.0449)	0.1185^***^ (0.0448)
Concern	0.1693^***^ (0.0570)	0.1539^***^ (0.0572)	0.1561^***^ (0.0569)	0.1447^**^ (0.0569)	0.1543^***^ (0.0571)
Lesson	0.1329 (0.1052)	0.1371 (0.1052)	0.1466 (0.1049)	0.1238 (0.1052)	0.1579 (0.1048)
Respect	0.3058^***^ (0.0645)	0.3583^***^ (0.0637)	0.3479^***^ (0.0641)	0.3572^***^ (0.0639)	0.3534^***^ (0.0645)
Legal	0.4777^***^ (0.0568)	0.4356^***^ (0.0540)	0.3581^***^ (0.0537)	0.4115^***^ (0.0561)	0.2945^***^ (0.0581)
Recogn	0.0693^*^ (0.0418)	0.1071^**^ (0.0416)	0.1009^**^ (0.0415)	0.0944^**^ (0.0415)	0.0914^***^ (0.0416)
Policy	0.1173^**^ (0.0537)	0.1359^**^ (0.0536)	0.1245^**^ (0.0537)	0.0959^*^ (0.0541)	0.1381^***^ (0.0534)
LR stat	282.0510^***^	275.9580^***^	254.5599^***^	264.4470^***^	235.6958^***^
Obs	1497	1497	1497	1497	1497

[1] ^***^,^**^ and^*^ denote significance level of 1, 5 and 10%, respectively; [2] Clustered standard error in parentheses

By introducing different legal environment variables and using stepwise regression technology in Table 6 and Table A2, the test results of H1b could be proved robust. For the legal environment in Table 6, the five variables had significant positive impact. Thus, although the legal environment does not improve the overall intention to practice medicine, it plays a role in shifting the intention to low-level hospitals in the medical market.

Regarding the policy effect of reform, Table 6 provides obvious evidence of positive correlation between the intention to practice in low-level hospitals and perceived reform effect. And *Recogn* also has a positive link with intention significantly, showing that students need not bear the reform costs if choosing to work at low-level hospitals.

### Impact of legal environment on reducing violence

Test result of H2b. As seen in Table 7, all legal environment variables caused a significant perceived decline in violence, confirming Hypothesis 2b. As shown in Table 5, *Violence* negatively impacted the willingness to practice medicine, and thus we inferred that an improvement in the legal environment could contribute to a decline in violence, and indirectly affect the working intention of medical students.

**Table 7:** OLM estimation results of perceived improvement in healthcare workplace violence

Dependent variable: perceived improvement in incidence of violence Y2 (1–5 variables from Very low, Low, Moderate, High, Very high)					
Variable	Model 1: Legal__A_	Model 2: Legal__B_	Model 3: Legal__C_	Model 4: Legal__D_	Model 5: Legal__E_
Target	0.8491^***^ (0.2246)	0.8391^***^ (0.2250)	0.7442^***^ (0.2266)	0.8335^***^ (0.2267)	0.8821^***^ (0.2272)
Education	0.4095^***^ (0.1038)	0.4004^***^ (0.1046)	0.4155^***^ (0.1041)	0.3934^***^ (0.1043)	0.3163^**^ (0.1046)
Grade	−0.1128^***^ (0.0386)	−0.1264^***^ (0.0386)	− 0.1297^***^ (0.0385)	− 0.1230^**^ (0.0386)	− 0.1383^***^ (0.0385)
Concern	0.2882^***^ (0.0574)	0.2524^***^ (0.0579)	0.2555^***^ (0.0573)	0.2562^***^ (0.0578)	0.2492^***^ (0.0578)
Legal	0.4975^***^ (0.0577)	0.4134^***^ (0.0548)	0.5315^***^ (0.0555)	0.5568^***^ (0.0574)	0.5193^***^ (0.0596)
Lesson	0.1871^*^ (0.1061)	0.2154^**^ (0.1059)	0.2061^*^ (0.1062)	0.1652 (0.1065)	0.1935 (0.1061)
Respect	0.1751^***^ (0.0637)	0.2246^***^ (0.0634)	0.1882^***^ (0.0632)	0.1952^***^ (0.0633)	0.1739^**^ (0.0640)
Recogn	0.0787^*^ (0.0423)	0.1148^***^ (0.0421)	0.1073^**^ (0.0422)	0.1033^**^ (0.0423)	0.0963^**^ (0.0422)
Policy	0.2787^***^ (0.0547)	0.3077^***^ (0.0545)	0.2672^***^ (0.0549)	0.2429^***^ (0.0553)	0.2794^***^ (0.0546)
LR stat	393.4340^***^	375.2681^***^	411.7182^***^	414.2574^***^	395.4193^***^
Obs	1497	1497	1497	1497	1497

[1] ^***^,^**^ and^*^ denote significance level of 1, 5 and 10%, respectively; [2] Clustered standard error in parentheses

Compared with our previous results (Tables 5 and 6), health policy education variable now significantly generates a positive link to perceived decline in violence. The reason could be that, although medical education itself cannot influence violent perpetrators, it can help students to truly understand the incidence of WPV and medical environment. Besides, it should be noted that the policy effect of reform also contributed to the perceived decline in the incidence of violence, indicating that descending resources reform has helped to rebalance patient trust, reduce congestion in the medical market, and decrease the WPV incidence.

## Discussion

The goal of medical education is to provide sustainable human capital supply for the medical market and different levels of hospitals. However, medical students are facing a variety of career choices other than practicing medicine, and their career choices depend on the satisfaction (utility) of different demand levels brought by practicing medicine compared with other occupations. If medical students expect that being a healthcare worker means higher safety risks and higher practice costs, their willingness to practice medicine will be weakened [47, 48], which will result in medium and long-term insufficient supply of medical services because of shortage of human capital. In the context of ongoing descending resources reform and high incidence of healthcare workplace violence, China’s dominant public hospital system and hospital classification system enable us to observe how medical students will respond to the reform and workplace violence, and help us understand the inter- and intra-market effect of exogenous shock on medical students’ willingness to practice medicine.

Our study confirmed that, the perceived violence incidence of medical students has a negative impact on their working intentions to practice medicine and a positive impact on their intentions to practice in low-level hospitals. The negative impact means the existence of inter-market effect, that is, the higher incidence of perceived violence means the expected higher practice risk and the lower satisfaction of safety needs compared with other occupations. Then, it will inevitably weaken the working intentions to practice medicine for medical students [4, 12]. At the same time, there exist obvious differences in the WPV incidence between different levels of hospitals, and high-level hospitals usually experience much higher incidence than that of low-level hospitals [31, 32]. So, medical students with factor specificity will more choose low-level hospital so as to receive higher satisfaction of safety needs in the medical market. Therefore, the positive effect of the perceived incidence of workplace violence reflects this intra-market effect, that is, the high WPV incidence in high-level hospitals will drive medical students to choose low-level hospitals.

China’s recent high WPV incidence is accompanied by the ongoing descending health resources reform. This reform is characterized by the fact that young doctors in high-level hospitals need to bear the reform cost by leaving cities where they work and descending to nonlocal low-level hospitals [49]. But at the same time, the goal of this reform is to alleviate the congestion of high-level hospitals and make better use of the idle resources of low-level hospitals, so as to correct the resource mismatch in China’s medical market. It will also help to improve the practice environment of medical students in the future and improve their utility level. Therefore, the effect of reform policy perceived by medical students will positively impact their willingness to practice medicine, but their cognition of reform means that their choice to practice medicine will make them bear the reform cost in the near future, which could exert a negative effect. Our estimation results support the coexistence of the above two opposite reform effects. However, since working in low-level hospitals means no need to bear the reform cost, the above negative effect of reform cognition will tend to disappear, which is also confirmed by our empirical results.

There may be concerns that the cross-sectional questionnaire data used in this paper cannot fully reflect students’ real career choice behavior after graduation. It should be noted that, medical students would not make decision on their career choice only around graduation period, their working intentions to practice medicine depend on the collection and evaluation of all available information in the whole process of medical education. Even if medical students’ cognition on the incidence of workplace violence and health reform seriously deviates from the real situation, they will make career choices based on it. However, in the case of biased information, medical students tend to hold a low willingness to practice medicine, which is against the goal of medical education and requires intervention in the early stage of medical education.

This study has several limitations. Firstly, due to the nature of the cross-sectional data and models used, our empirical results should not be interpreted in terms of causality, but rather as correlations. In addition, working intention does not fully determine real career choice when students graduate and enter the labor market. If follow-up investigation could be performed, we can combine the real career choice data with the working intention response data to the model, which would be helpful for us to better understand the dynamic impact of exogenous shock on medical students’ career choice.

## Conclusions

Workplace violence against healthcare workers is a global problem [37, 50], and some reports have found that such violence occurs in medical students and impact their working intention to practice medicine [48, 51]. This paper focused on the impact of WPV and the anti-violence legal environment on medical students’ working intentions in the context of China’s descending resources reform launched in 2013. We identified inter- and intra-market effects of WPV on medical students’ career choice, emphasizing risk aversion motives and the importance of medical student safety when facing severe WPV risk. This research provides new insights into the factors influencing medical students’ career choices beyond family, personality, and academic achievement.

Based on our results, the anti-violence legal environment has no direct link with medical students’ working intentions but may contribute to the perceived decline in the incidence of violence. Our study also showed that the perceived costs and benefits of reform have the opposite effects on driving students’ desire to practice medicine, thereby highlighting the complexity of reform. Thus, the anti-violence environment and reform policies may impact medical students, and generate an intra-market effect, i.e., push medical students to work in low-level hospitals. To incentivize constant inflow and balanced allocation of human capital in the health market, urgent action is needed to reduce the incidence of healthcare WPV with the cooperation of the government, legislation, law enforcement, and medical institutions. Improving student awareness of WPV and reform could also encourage more rational and well-informed career choices.

## Supplementary Information

**Additional file 1.**

## Data Availability

The datasets used and/or analyzed during the current study available from the corresponding author on reasonable request.

## Abbreviations

OLM: Ordered logit model
WPV: Healthcare workplace violence
KMO: Kaiser-Meyer-Olkin test

## References

[1] Hesketh T, Wu D, Mao L, Ma N (2014). Violence against doctors in China. Br Med J.

[2] Lancet T (2020). Protecting Chinese doctors. Lancet..

[3] Chai PP, Zhang YH, Zhou MG, Liu SW, Kinfu Y (2020). Health system productivity in China: a comparison of pre- and post-2009 healthcare reform. Health Policy Plann..

[4] Sun ZS, Wang SH, Zhao HJ, Yu HM (2020). Does descending resources reform improve patient satisfaction and reshape choice of care providers? A cross-sectional study in Zhejiang, China. Inquiry.

[5] Guan J (2017). Origin and prevention of workplace violence in health care in China: legal and ethical considerations. Chinese Med J.

[6] Zheng JW (2017). Deepening the reform of medical and health systems and promoting the construction of a healthy Zhejiang. Admin Reform.

[7] Patterson F, Roberts C, Ponnamperuma GG (2018). 2018 Ottawa consensus statement: selection and recruitment to the healthcare professions. Med Teach.

[8] Girotti J, Park YS, Tekian A (2015). Ensuring a fair and equitable selection of students to serve society’s healthcare needs. Med Educ.

[9] Kazi AS, Akhlaq A (2017). Factors affecting career choice. J Res Reflect Educ.

[10] Jackson D, Clare J, Mannix J (2002). Who would want to be a nurse? Violence in the workplace-a factor in recruitment and retention. J Nurs Manage.

[11] Scott A, Joyce C, Chen T (2013). Medical career path decision making: an evidence check rapid review. Sax Institute for the NSW Ministry of Health Australia.

[12] Warshawski S (2021). Workplace violence directed at nursing and medical students-what can students tell us about it?. J Prof Nurs.

[13] Tee S, Ozcetin YSU, Russell-Westhead M (2016). Workplace violence experienced by nursing students: a UK survey. Nurse Educ Today.

[14] Shapiro J, Boyle MJ, McKenna L (2018). Midwifery student reactions to workplace violence. Women Birth.

[15] Furst C (2018). The relationship between experiences of lateral violence and career choice satisfaction among nursing students. Nurs Educ Perspect.

[16] Han X, Wang Y, Zhao J (2014). Examining influence of violence against physicians on Chinese medical students’ career choice. Chin Med J.

[17] Sun ZS, Barnes SR, Wang SH (2016). Understanding congestion in China’s medical market: an incentive structure perspective. Health Policy Plann..

[18] Tang WJ. Survey repeals applicants of medical students dropped. Wen Hui Bo. 2011; http://fashion.ifeng.com/health/disease/detail_2011_10/17/9905767_0.shtml.

[19] Wang CC. Medical graduates have the lowest average salary and top high school students do not want to study medicine. China Youth Daily. 2013; Available from: http://finance.people.com.cn/n/2013/0523/c1004-21581902.html. Accessed 16 Sept 2020.

[20] Hu F (2014). Survey: 94% respondents do not want their children to pursue a medical career.

[21] Ye C, Cai SZ, Hu SX, Yu F, Dong LL (2019). Investigation of medical personnel’s children’s intentions to pursue medical careers in a tertiary hospital in Wuhan. Med Soc.

[22] Chen SM, Ren C, Chen BX, Feng XH, Zeng H, Gao W (2015). The influence of violence against medical practitioners on high school students’ intentions to practice medicine. China High Med Educ.

[23] Lin SZ, Chai WL, Lin XR, Li DL, Hong RY, Wu SY (2014). Analysis of influential factors of doctor's turnover intention. Mod Prevent Med.

[24] Borracci RA, Arribalzaga EB, Couto JL, Dvorkin M, Cerezo L (2015). Factors affecting willingness to practice medicine in underserved areas: a survey of argentine medical students. Rural Remote Health.

[25] Ossai E, Anyanwagu U, Azuogu B, Uwakwe K, Ibiok N (2015). Student perception about working in rural area after graduation and associated factors: a study among final year medical students in medical schools of Southeast Nigeria. Br J Med & Med Res.

[26] Rezaei S, Hajizadeh M, Karyani AK, Soltani S, Asadi H, Bazyar M, Mohammadi Z (2019). Determinants of willingness to practice medicine in underdeveloped areas: evidence from a survey on Iranian medical students. Clin Gov.

[27] Zhang L, Bossert T, Mahal A, Hu G, Guo Q, Liu YL (2016). Attitudes towards primary care career in community health centers among medical students in China. BMC Fam Pract.

[28] Tian LL, Zhu J, Sun ZY, Chen YM, Zhang LD, Wang TY, Hu D (2020). Medical students’ working intentions in grassroot hospitals and its influencing factor. Rural Health Admin China.

[29] Hou JL, Wang WM, Ke Y (2018). Study of undergraduates’ willingness to practice clinical medicine and their post-graduation arrangements. Chinese Health Serv Manage.

[30] Li XX, Yuan T, Xing X, Zhang JY, Gu WJ, Yin M, Cheng ZY (2016). Investigation and factor analysis of hunting intention for rural grassroots of medical students in Gansu Province. Chinese J Health Policy.

[31] Chen W, Wang XL (2016). Big data decryption of the general rules of violence that caused wounding in the medical profession.

[32] Zhao M, Jiang YM, Yang LL, Qu WY (2017). Big data research on violent injury cases—based on media reports from 2000 to 2015. Med Philos.

[33] WHO, World Bank (2016). Deeping health reform in China: building high-quality and value-based service delivery.

[34] Meng Q, Mills A, Wang L, Han Q (2019). What can we learn from China’s health system reform?. Br Med J.

[35] Xi JP (2014). Prosperity for all is impossible without health for all. China Central Television.

[36] Zhejiang Province Government (2015). Implementation opinions on promoting the construction of long-term mechanism for reform of double descendings and two promotions.

[37] Phillips JP (2016). Workplace violence against health care workers in the United States. New Engl J Med.

[38] Rhoades KA, Heyman RE, Eddy JM, Fat SJ, Haydt NC, Glazman JE, Dispirito ZF (2020). Patient aggression toward dental students. J Dent Educ.

[39] Afkhamzadeh A, Mohamadi BA, Moloudi B, Safari H, Piroozi B (2019). Workplace violence against physicians and medical students in west part of Iran. Int J Hum Rights Healthc.

[40] Wang N, Yang YZ, Wu D, Li L, Zhou XD, Yang Q (2018). Study of violence against the nursing staff and their tolerance in Zhejiang province. J Nurs Sci.

[41] Meng LM, Lin DM, Lai Y, Huang SM, Ying H, Zhao YL, Zhong HQ (2019). Influence of experience and knowledge of hospital violence on medical students’ professional identify of a university in Ganzhou city. Med Soc.

[42] Lin WJ, Shi L, Tang PY, Liu J (2017). The effect of media reports of violence injury medical events on medical students. Chinese Med Ethics.

[43] Hong D, Huang F, Zeng W, Huang C, Liu XH (2015). Influence of hospital violence events on medical students. Chinese Nurs Res.

[44] Robbins SP (1988). Management: concepts and applications.

[45] Ding JF, Liu QY (2018). The cause and coping strategies of healthcare workplace violence under the perspective of Maslow’s hierarchy of needs. J Nanjing Medi Univ (Soc Sci).

[46] Pan J, Liu D, Ali S (2015). Patient dissatisfaction in China: what matters. Soc Sci Med.

[47] Wang S, Sun Z (2020). A survey on the recognition and working intention responses of medical students to sinking resources reform. J Chinese Med Educ.

[48] Leaune E, Rey-Cadihac V, Oufker S, Grot S, Strowd R, Rode G, Crandall S (2021). Medical students attitudes toward and intention to work with the underserved: a systematic review and meta-analysis. BMC Med Educ.

[49] Sun Z, Wang S, Zhao H, Zhou X, Zhang L, Shi J (2021). Does descending health resources reform impact low-level hospital selection behavior? evidence from Zhejiang, China. Arch Public Health.

[50] Nelson R (2014). Tackling violence against health-care workers. Lancet..

[51] Boyle MJ, McKenna L (2016). Paramedic and midwifery student exposure to workplace violence during clinical placements in Australia-a pilot study. Int J Med Educ.

