# Supplementary material for: Working intentions of medical students in response to healthcare workplace violence and descending resources reform in China
Source: BMC Med Educ. 2022 May 9;22:351. doi: 10.1186/s12909-022-03428-1 (PMC9088100; doi:10.1186/s12909-022-03428-1)
Supplement: Supplementary file 1 — Additional file 1. [file 12909_2022_3428_MOESM1_ESM.docx]

**Appendix**

**Table A1** Stepwise OLM regression results of medical students’ working intention

| Dependent variable: working intention Y (1 for decrease, 2 for no change, 3-5 for slight increase, significant increase, and great increase) | | | | | | | |
| --- | --- | --- | --- | --- | --- | --- | --- |
| Variable | 1 | 2 | 3 | 4 | 5 | 6 | 7 |
| Target | 0.8842^***^  (0.2110) | 0.8146^***^  (0.2100) | 0.8555^***^  (0.2115) | 0.8448^***^  (0.2122) | 0.8454^***^  (0.2134) | 0.8711^***^  （0.2146） | 0.8102^***^  （0.2150） |
| Education | 0.7243^***^  (0.0977) | 0.6196^***^  (0.0990) | 0.6462^***^  (0.1003) | 0.6413^***^  (0.1006) | 0.6415^***^  (0.1008) | 0.6931^***^  （0.1029） | 0.6571^***^  （0.1031） |
| Grade | -0.1297^**^  (0.0367) | -0.1158^**^  (0.0368) | -0.1107^***^  (0.0369) | -0.1090^***^  (0.0369) | -0.1091^***^  (0.0371) | -0.1030^**^  （0.0372） | -0.0934^**^  （0.0373） |
| Violence | -0.4752^***^  (0.0453) | -0.4679^***^  (0.0454) | -0.4622^***^  (0.0455) | -0.4604^***^  (0.0455) | -0.4604^***^  (0.0456) | -0.4502^***^  （0.0456） | -0.4389^***^  （0.0456） |
| Concern |  | 0.2631^***^  (0.0536) | 0.2719^***^  (0.0539) | 0.2671^***^  (0.0543) | 0.2671^***^  (0.0544) | 0.2776^***^  （0.0547） | 0.2329^***^  （0.0558） |
| Lesson |  |  | -0.1745^*^  (0.1024) | -0.1810^*^  (0.1028) | -0.1807^*^  (0.1034) | -0.1249  （0.1055） | -0.1518  （0.1060） |
| Respect |  |  |  | 0.0410  (0.0596) | 0.0415  (0.0616) | 0.0454  （0.0618） | 0.0186  （0.0622） |
| Legal__A_ |  |  |  |  | -0.0015  (0.0530) | -0.0201  （0.0538） | -0.0166  （0.0548） |
| Recogn |  |  |  |  |  | -0.1120^***^  （0.0423） | -0.1322^***^  （0.0499） |
| Policy |  |  |  |  |  |  | 0.2269^***^  （0.0545） |
| LR stat | 268.4967 | 292.8147^***^ | 295.7275^***^ | 296.2013^***^ | 296.2021^***^ | 303.2629^***^ | 320.6237^***^ |
| Obs | 1497 | 1497 | 1497 | 1497 | 1497 | 1497 | 1497 |

Note: [1] ^***^,^**^ and^*^ denote significance level of 1%, 5% and 10%, respectively; [2] Clustered standard error in parentheses.

**Table A2** Stepwise OLM regression results of medical students’ intention to work in low-level hospitals

| Dependent variable: working intention for low-level hospital Y1 (1 for decrease, 2 for no change, 3-5 for slight increase, significant increase, and great increase) | | | | | | | |
| --- | --- | --- | --- | --- | --- | --- | --- |
| Variable | 8 | 9 | 10 | 11 | 12 | 13 | 14 |
| Target | 1.2269^***^  (0.2209) | 1.1364^***^  (0.2210) | 1.0823^***^  (0.2218) | 0.9955^***^  (0.2226) | 0.8343^***^  (0.2232) | 0.8292^***^  （0.2235） | 0.7949^***^  （0.2242） |
| Education | 0.8068^***^  (0.0997) | 0.6780^***^  (0.1025) | 0.6349^***^  (0.1033) | 0.5578^***^  (0.1035) | 0.4888^***^  (0.1035) | 0.4508^***^  （0.1055） | 0.4278^***^  （0.1062） |
| Grade | -0.0371  (0.0376) | -0.0237  (0.0377) | -0.0345  (0.0379) | -0.0186  (0.0380) | 0.0163  (0.0383) | 0.0130  （0.0384） | 0.0165  （0.0384） |
| Violence | 0.0751^*^  (0.0441) | 0.0895^**^  (0.0441) | 0.0820^*^  (0.0442) | 0.1038^**^  (0.0443) | 0.1275^***^  (0.0445) | 0.1206^***^  （0.0446） | 0.1285^***^  （0.0449） |
| Concern |  | 0.2855^***^  (0.0554) | 0.2723^***^  (0.0556) | 0.2299^***^  (0.0558) | 0.2005^***^  (0.0557) | 0.1943^***^  （0.0558） | 0.1693^***^  （0.0570） |
| Lesson |  |  | 0.2948^***^  (0.1021) | 0.2436^***^  (0.1026) | 0.1800^*^  (0.1034) | 0.1463  （0.1050） | 0.1329  （0.1052） |
| Respect |  |  |  | 0.4510^***^  (0.0633) | 0.3257^***^  (0.0642) | 0.3218^***^  （0.0642） | 0.3058^***^  （0.0645） |
| Legal__A_ |  |  |  |  | 0.5046^***^  (0.0562) | 0.4917^***^  （0.0566） | 0.4777^***^  （0.0568） |
| Recogn |  |  |  |  |  | 0.0776^*^  （0.0416） | 0.0693^*^  （0.0418） |
| Policy |  |  |  |  |  |  | 0.1173^**^  （0.0537） |
| LR stat | 104.0532^***^ | 130.8074^***^ | 295.7275^***^ | 296.2013^***^ | 273.7907^***^ | 277.2709^***^ | 282.0510^***^ |
| Obs | 1497 | 1497 | 1497 | 1497 | 1497 | 1497 | 1497 |

Note: [1] ^***^,^**^ and^*^ denote significance level of 1%, 5% and 10%, respectively; [2] Clustered standard error in parentheses.
